# High serum folate level is positively associated with pulmonary function in elderly Korean men, but not in women

**DOI:** 10.1038/s41598-022-08234-9

**Published:** 2022-03-16

**Authors:** Suk Won Chang, Min Bum Kim, Ju Wan Kang

**Affiliations:** 1 Department of Otorhinolaryngology, Jeju National University College of Medicine, Jeju, Republic of Korea; 2 Department of Otorhinolaryngology, Gangnam Severance Hospital, Yonsei University College of Medicine, 2111, Eonju-ro, Gangnam-gu, Seoul, 06237 Republic of Korea

**Keywords:** Nutrition, Respiratory signs and symptoms

## Abstract

A limited number of studies have been conducted on the relationship between serum vitamin levels and pulmonary function, particularly in the elderly population. This study attempted to confirm the association between serum vitamin levels (folate, vitamin A, and vitamin E) and pulmonary function in the elderly population of Korea. A total of 1166 subjects (528 men and 637 women) participated in the Korean National Health and Nutrition Examination Survey from 2016 to 2018. Serum levels of folate, vitamin A, and vitamin E were measured in the subjects. The subjects’ pulmonary function measurement items were as follows: forced vital capacity (FVC), forced expiratory volume in one second (FEV1), forced expiratory volume in one second/forced vital capacity (FEV1/FVC), forced expiratory flow at 25% and 75% of the pulmonary volume (FEF25–75%), forced expiratory volume in 6 s (FEV6), and peak expiratory velocity (PEV). We performed regression analysis considering FEV1, PEV, FVC, FEF25–75%, and FEV1/FVC and FEV6 as dependent variables. Serum vitamin A levels were not associated with pulmonary function. In elderly men, serum vitamin E levels were negatively correlated with FVC [B =  − 0.012, 95% confidence interval (CI) − 0.022 to − 0.003, *p* = 0.012] and FEV1 (B =  − 0.010, 95% CI − 0.115 to − 0.007, *p* = 0.028). We confirmed a positive correlation of the serum folate level with FEV1 (B = 0.017, 95% CI 0.004–0.030, *p* = 0.009), FEV1/FVC (B = 0.003, 95% CI 0.001–0.005, *p* = 0.007), and FEF25–75% (B = 0.031, 95% CI 0.010–0.053, *p* = 0.005) in elderly men. This study confirmed that high serum folate levels were positively associated with pulmonary function in elderly men in Korea. Further studies are needed to understand the longitudinal effect of folate and its biological mechanism in pulmonary function.

## Introduction

The improving economic levels and rising interest in health-related vitamins in the modern society has resulted in an increased intake of vitamins in adults^1^. As a result, sales of supplements such as vitamins has steadily increased to 30 billion dollars, and has a widespread impact on public health in the United States^2^. Among these supplements, antioxidant vitamins such as vitamin C and vitamin E have been reported to capture organic free radicals and reduce cardiovascular disease by preventing the formation of atherosclerotic plaques^3^. Additionally, an inverse relationship has been observed between the incidence of type 2 diabetes and vitamin levels^4^. In addition, a significant difference in glycated hemoglobin (HbA1c) levels has been observed between the group taking vitamins C and E and the group not taking vitamins C and E^5^.

Studies have revealed that an increased intake of antioxidant vitamins, such as vitamin C, can improve pulmonary function and protect human lungs^6,7^. In particular, it has been reported that low antioxidant vitamin intake increases the risk of chronic constructive pulmonary disease (COPD), especially in men who smoke^8^. Recently, several studies have further shown the association between folate and pulmonary function. A study by Jung et al. reported that a lack of folate intake was associated with the development of airflow limitation^9^. In addition, a previous study has indicated that the intake of appropriate folates may reduce the incidence of airway diseases such as COPD^10^.

However, only a few studies have reported the association between various serum vitamins and pulmonary function in the elderly population. Therefore, this study attempted to analyze the association between various serum vitamins and pulmonary function in the elderly population aged 60 to 80 years using data from the Korean National Health and Nutrition Examination Survey (KNHANES) (2016–2018). In particular, this study attempted to analyze the association between serum folate, vitamin A, and vitamin E levels and pulmonary function.

## Results

We analyzed a total of 1,166 elderly individuals aged 60–80 years (528 men and 637 women) in this study. There was no significant difference in the mean age between men (68.1 ± 5.9) and women (68.3 ± 6.3). The mean serum levels of folate [7.1 (± 3.5) vs. 9.1 (± 3.9); *p* < 0.001] and vitamin E [13.9 (± 5.2) vs. 15.2 (± 5.8); *p* < 0.001] were significantly higher in women than in men. On the contrary, the mean serum levels of vitamin A were higher in men than in women [13.9 (± 5.2) vs. 15.2 (± 5.8); *p* < 0.001]. The Cohen’s D values of folate, vitamin A, and vitamin E were 0.527, 0.408, and 0.247, respectively. Therefore, it is considered that the value of folate was a relatively larger difference between men and women compared to vitamin A and vitamin E. The mean values of the pulmonary function test items for all participants are as follows: FVC: 3.1 (± 0.8) L, FEV1: 2.3 (± 0.6) L, FEV1/FVC: 0.7 (± 0.1), FEF 25–75%: 1.9 (± 0.8) L/second, FEV6: 2.9 (± 0.7) L, PEV: 6.0 (± 1.9) L/min. The mean FVC, FEV1, FEV6, and PEV values were higher in men than in women (Table 1).

**Table 1:** Demographic data, clinical parameters, serum folate, vitamin values, and pulmonary function of subjects.

	Total (N = 1166)	Men (n = 528)	Women (N = 637)	*p* value
Age (years)^$^	68.2 ± 6.1	68.1 ± 5.9	68.3 ± 6.3	0.521
Height (cm)^$^	158.8 ± 8.8	167.2 ± 5.8	153.7 ± 5.7	< 0.001
Weight (Kg)^$^	62.3 ± 9.8	67.6 ± 5.8	57.9 ± 8.0	< 0.001
Alcohol Hx*				
No	244	40	204	< 0.001
Yes	922	489	433	
**Smoking Hx**^**#**^				
No	703	99	604	< 0.001
less than 5packs	9	8	1	
More than 5 packs	454	422	32	
Folate (ng/mL)^$^	8.2 ± 3.8	7.12 ± 3.46	9.06 ± 3.87	< 0.001
Vitamin A (mg/L)^$^	0.5 ± 0.2	0.59 ± 0.20	0.51 ± 0.17	< 0.001
Vitamin E (mg/L)^$^	14.6 ± 5.6	13.86 ± 5.16	15.23 ± 5.83	< 0.001
FVC (L)^$^	3.1 ± 0.8	3.67 ± 0.69	2.54 ± 0.46	< 0.001
FEV1 (L)^$^	2.3 ± 0.6	2.60 ± 0.61	1.97 ± 0.38	< 0.001
FEV1/FVC (%)^$^	0.7 ± 0.1	0.71 ± 0.9	0.77 ± 0.06	< 0.001
FEF25–75% (L/s)^$^	1.9 ± 0.8	1.93 ± 0.93	1.88 ± 0.67	0.240
FEV6 (L)^$^	2.9 ± 0.7	3.47 ± 0.67	2.49 ± 0.44	< 0.001
PEV (L/s)^$^	6.0 ± 1.9	7.21 ± 1.99	5.03 ± 1.19	< 0.001

Demographic data of study population were presented as appropriate.

Alcohol history and smoking history—number of each group; Height, weight, folate, vitamin A, vitamin E, FVC, FEV1, FEV/FVC, FEF25–75%, FEV6, PEV—presented with mean ± standard deviation.

*Using chi square test.

^#^Using linear by linear association.

^$^Using student t-test.

We performed unadjusted linear regression analysis between each pulmonary function test and each serum vitamin level. Results showed very different results between each vitamin and lung function results depending on the target group. (Supplemental Tables 1, 2, and 3). Therefore, we decided to include three vitamins and variables such as weight, height, and smoking history, which were known to be correlated with lung function in previous studies, in multivariate linear regression.

**Table 2:** Multivariate linear regression analysis considering FVC as dependent variable.

	Total			Men			Women		
	B	95% CI	*p*	B	95% CI	*p*	B	95% CI	*p*
Sex	− ***0.468***	− ***0.574*** to − ***0.362***	< ***0.001***						
Age	− ***0.028***	− ***0.033*** to − ***0.024***	< ***0.001***	− ***0.035***	− ***0.043*** to − ***0.027***	< ***0.001***	− ***0.025***	− ***0.030*** to − ***0.020***	< ***0.001***
Alcohol Hx	0.038	− 0.031 to 0.108	0.280	0.125	− 0.053 to 0.304	0.168	0.042	− 0.020 to 0.104	0.189
Smoking Hx	0.011	− 0.032 to 0.054	0.620	0.013	− 0.046 to 0.073	0.662	0.012	− 0.052 to 0.076	0.716
Height	***0.047***	***0.042*** to ***0.053***	< ***0.001***	***0.061***	***0.051*** to ***0.071***	< ***0.001***	***0.037***	***0.031*** to ***0.042***	< ***0.001***
Weight	− 0.001	− 0.005 to 0.002	0.504	− 0.002	− 0.008 to 0.004	0.597	− ***0.004***	− ***0.008*** to ***0.000***	***0.047***
Folate	0.000	− 0.007 to 0.008	0.937	0.009	− 0.005 to 0.022	0.231	− 0.004	− 0.011 to 0.004	0.331
Vitamin A	− 0.062	− 0.207 to 0.083	0.401	− 0.138	− 0.370 to 0.095	0.246	− 0.027	− 0.195 to 0.141	0.755
Vitamin E	− 0.003	− 0.008 to 0.002	0.215	− ***0.012***	− ***0.022*** to − ***0.003***	***0.012***	0.003	− 0.002 to 0.008	0.275

Bold italics indicates statistical significance (*p* < 0.05).

*CI* Confidence interval.

**Table 3:** Multivariate linear regression analysis considering FEV1 as dependent variable.

	Total			Men			Women		
	B	95% CI	*p*	B	95% CI	*p*	B	95% CI	*p*
Sex	− ***0.313***	− ***0.406*** to − ***0.220***	< ***0.001***						
Age	− ***0.031***	− ***0.035*** to − ***0.027***	< ***0.001***	− ***0.040***	− ***0.047*** to − ***0.033***	< ***0.001***	− ***0.025***	− ***0.029*** to − ***0.021***	< ***0.001***
Alcohol Hx	0.031	− 0.030 to 0.093	0.319	0.114	− 0.048 to 0.276	0.169	0.031	− 0.021 to 0.082	0.240
Smoking Hx	− ***0.057***	− ***0.094*** to− ***0.019***	***0.003***	− ***0.061***	− ***0.115*** to − ***0.007***	***0.028***	− 0.035	− 0.088 to 0.018	0.193
Height	***0.026***	***0.021*** to ***0.031***	< ***0.001***	***0.031***	***0.023*** to ***0.040***	< ***0.001***	***0.022***	***0.018*** to ***0.027***	< ***0.001***
Weight	***0.005***	***0.002*** to ***0.008***	***0.003***	***0.006***	***0.001*** to ***0.012***	***0.025***	0.000	− 0.003 to 0.003	0.818
Folate	0.004	− 0.002 to 0.011	0.173	***0.017***	***0.004*** to ***0.030***	***0.009***	− 0.003	− 0.009 to 0.004	0.412
Vitamin A	− 0.020	− 0.108 to 0.147	0.763	− 0.018	− 0.230 to 0.193	0.864	0.006	− 0.133 to 0.144	0.934
Vitamin E	− 0.003	− 0.007 to 0.002	0.220	− ***0.010***	− ***0.115*** to − ***0.007***	***0.028***	0.002	− 0.002 to 0.006	0.361

Bold italics indicates statistical significance (*p* < 0.05).

*CI* Confidence interval.

When considering FVC as a dependent variable, the serum folate level did not show a significant difference in men [B = 0.009, 95% confidence interval (CI) − 0.005 to 0.022, *p* = 0.231] and women (B =  − 0.004, 95% CI − 0.011 to 0.004, *p* = 0.331). However, with respect to the serum vitamin E level, a negative correlation was observed in men (B =  − 0.012, 95% CI − 0.022 to − 0.003, *p* = 0.012) (Table 2). When FEV1 was considered as a dependent variable, we confirmed that the serum folate level was positively correlated in men (B = 0.017, 95% CI 0.004–0.030, *p* = 0.009), while the serum vitamin E level was negatively correlated with FEV1 (B =  − 0.010, 95% CI − 0.115 to − 0.007, *p* = 0.028) (Table 3). When FEV1/FVC was considered as a dependent variable, we confirmed that serum folate level was positively correlated in men (B = 0.003, 95% CI 0.001–0.005, *p* = 0.007) (Table 4). When FEF 25–75% was considered as a dependent variable, the serum folate level tended to be positively correlated in men (B = 0.031, 95% CI 0.010–0.053, *p* = 0.005) (Table 5). When PEV was considered as a dependent variable, serum folate level showed a positive correlation in men (B = 0.072, 95% CI 0.027–0.118, *p* = 0.002) (Supplemental Table 1). When FEV6 was considered as a dependent variable, serum vitamin E level showed a negative correlation in men (B =  − 0.012, 95% CI − 0.021 to − 0.03, *p* = 0.009) (Supplemental Table 2).

**Table 4:** Multivariate linear regression analysis considering FEV1/FVC as dependent variable.

	Total			Men			Women		
	B	95% CI	*p*	B	95% CI	*p*	B	95% CI	*p*
Sex	***0.019***	***0.002*** to ***0.036***	***0.029***						
Age	− ***0.003***	− ***0.004*** to − ***0.003***	< ***0.001***	− ***0.004***	− ***0.006*** to − ***0.003***	< ***0.001***	− ***0.002***	− ***0.003*** to − ***0.002***	< ***0.001***
Alcohol Hx	0.002	− 0.009 to 0.013	0.735	0.010	− 0.020 to 0.039	0.523	0.001	− 0.009 to 0.011	0.797
Smoking Hx	− ***0.019***	− ***0.026*** to − ***0.012***	< ***0.001***	− ***0.020***	− ***0.030*** to − ***0.010***	< ***0.001***	− ***0.017***	− ***0.027*** to − ***0.006***	***0.001***
Height	− ***0.003***	− ***0.004*** to − ***0.002***	< ***0.001***	− ***0.003***	− ***0.005*** to − ***0.002***	< ***0.001***	− ***0.002***	− ***0.003*** to − ***0.001***	< ***0.001***
Weight	***0.002***	***0.001*** to ***0.002***	< ***0.001***	***0.002***	***0.001*** to ***0.003***	< ***0.001***	***0.001***	***0.001*** to ***0.002***	< ***0.001***
Folate	***0.001***	***0.000*** to ***0.002***	***0.032***	***0.003***	***0.001*** to ***0.005***	***0.007***	0.000	− 0.001 to 0.001	0.857
Vitamin A	0.020	− 0.003 to 0.043	0.090	0.025	− 0.013 to 0.063	0.204	0.009	− 0.018 to 0.036	0.511
Vitamin E	0.000	− 0.001 to 0.001	0.673	− 0.001	− 0.002 to 0.001	0.517	0.000	− 0.001 to 0.001	0.940

Bold italics indicates statistical significance (*p* < 0.05).

*CI* Confidence interval.

**Table 5:** Multivariate linear regression analysis considering FEF 25–75% as dependent variable.

	Total			Men			Women		
	B	95% CI	*p*	B	95% CI	*p*	B	95% CI	*p*
Sex	− ***0.194***	− ***0.362*** to − ***0.027***	***0.023***						
Age	− ***0.048***	− ***0.055*** to − ***0.041***	< ***0.001***	− ***0.054***	− ***0.066*** to − ***0.041***	< ***0.001***	− ***0.044***	− ***0.053*** to − ***0.036***	< ***0.001***
Alcohol Hx	0.015	− 0.095 to 0.126	0.787	0.108	− 0.169 to 0.386	0.443	− 0.005	− 0.111 to 0.101	0.931
Smoking Hx	− ***0.161***	− ***0.229*** to − ***0.093***	< ***0.001***	− ***0.169***	− ***0.262*** to ***0.076***	< ***0.001***	− ***0.129***	− ***0.238*** to ***0.020***	***0.021***
Height	− 0.003	− 0.011 to 0.006	0.560	− 0.002	− 0.016 to 0.013	0.836	− 0.002	− 0.012 to 0.007	0.658
Weight	***0.013***	***0.007*** to ***0.019***	< ***0.001***	***0.013***	***0.004*** to ***0.023***	***0.006***	***0.010***	***0.004*** to ***0.017***	***0.002***
Folate	0.008	− 0.004 to 0.020	0.173	***0.031***	***0.010*** to ***0.053***	***0.005***	− 0.006	− 0.019 to 0.006	0.340
Vitamin A	0.141	− 0.088 to 0.371	0.227	0.139	− 0.222 to 0.501	0.449	0.108	− 0.179 to 0.395	0.461
Vitamin E	− 0.001	− 0.009 to 0.007	0.863	− 0.008	− 0.023 to 0.007	0.302	0.003	− 0.006 to 0.011	0.546

Bold italics indicates statistical significance (*p* < 0.05).

*CI* Confidence interval.

**Figure 1:**
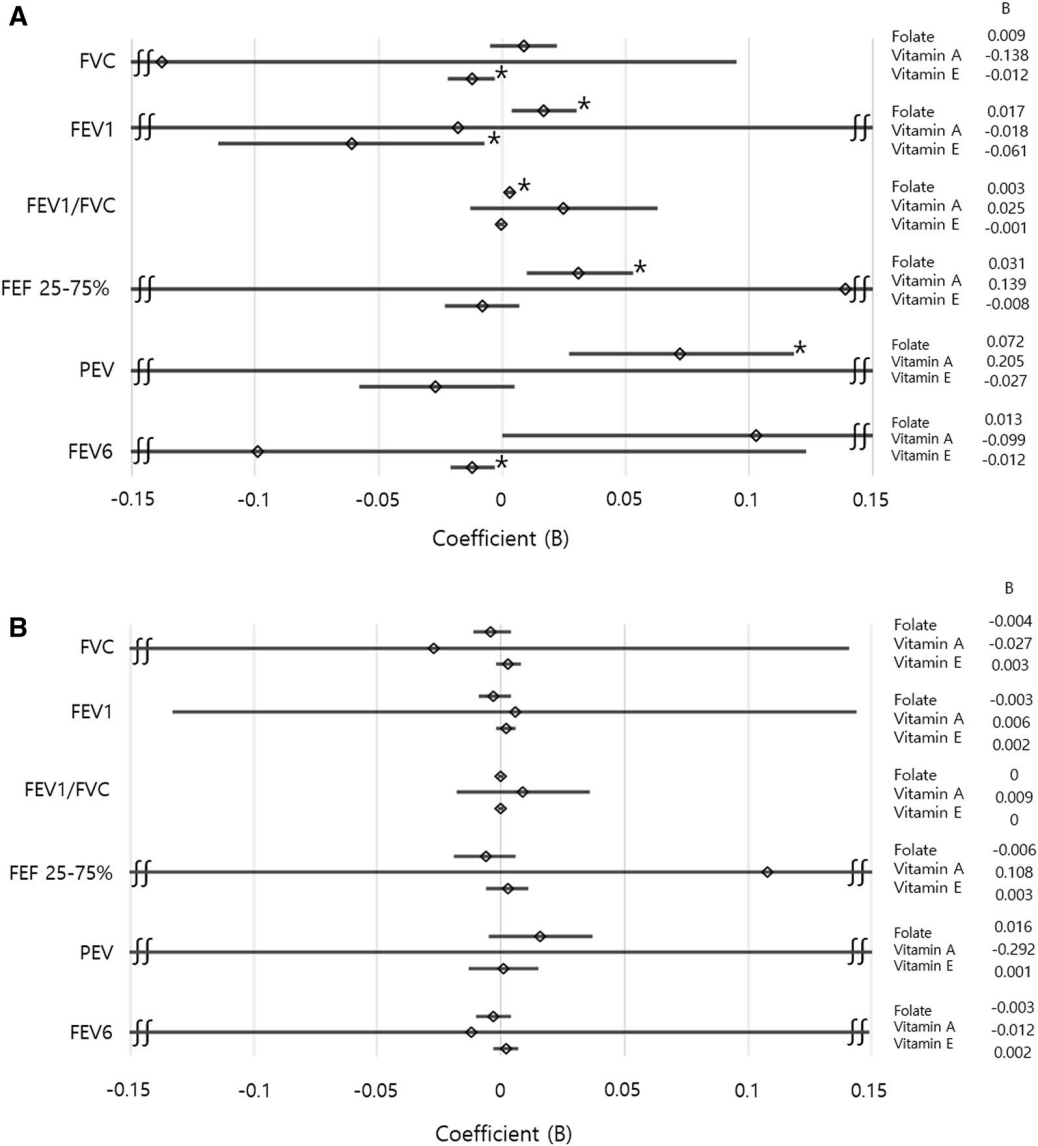
Multivariate linear regression analysis of the effects of serum vitamin levels on pulmonary function in men (**A**) and women (**B**) adjusted for age, alcohol history, smoking history, height, weight, and each serum vitamin level. *p < 0.05.

Figure 1 summarizes the correlation between serum vitamin level and lung function using multivariate linear regression analysis.

## Discussion

In this study, using KNHANES (2016–2018) data and measuring the pulmonary function, we confirmed that there is a positive association between serum folate level and various pulmonary function parameters in older men aged 60–80 years, but not in older women. In particular, serum folate level and FEV1, FEV1/FVC, and FEF 25–75%, which are considered important parameters for evaluating pulmonary function, were positively correlated. To the best of our knowledge, this is the first study to evaluate the relationship between pulmonary function and serum folate levels in the elderly population of Korea. Additionally, no positive association was observed between serum vitamin A and vitamin E levels, and pulmonary function in this study.

Therefore, the results of this study indicated that folate affected the FEV1, FEV1/FVC, and FEF25–75% of pulmonary function associated with obstructive ventilation disorders rather than FVC associated with restricted ventilation disorders in men. In addition, among the subjects included in this study, the proportion of smokers was higher in men than in women, which suggests that folate could help improve obstructive pulmonary function disorders caused by smoking. These results are similar to those of a previous study using KNHANES, which revealed that folate is associated with increased lung functions such as FEV1 in male smokers with COPD^14^.

Folate is a type of water-soluble vitamin B and is present in many leafy vegetables such as spinach, lettuce, and asparagus^15^. Folate plays an essential role in DNA methylation, hemoglobin synthesis, normal cell growth, replication, and brain development^16^. Further, the lack of folate is significantly associated with chronic diseases such as cardiovascular disease, depression, and Alzheimer's disease and causes birth defects such as natural tube defects in fetuses^11,17^. Several studies have shown the association between serum folate level and pulmonary function in airway diseases such as COPD or asthma. In a pediatric study, folate deficiency had a negative effect on pulmonary function in pediatric asthma patients^18^. Another study showed that asthma and high serum folate levels have a positive association in adults^19^.

This study confirmed that the serum folate level has a positive association with parameters that measure pulmonary function, such as FEV1, FEV1/FVC, and FEF 25–75%. In particular, FEV1 and FEV1/FVC have a tendency to decrease in patients with obstructive lung disease and are used as a diagnostic criteria for COPD, which is considered important in the pulmonary function test^11^. Previous studies have shown that in male COPD patients who smoke, high serum folate levels are positively correlated with pulmonary functions such as FEV1, FVC, and PEV^14^. Another previous study reported that among children, folate deficiency had a negative effect on pulmonary function in girls with asthma^18^. Similarly, Han et al. reported a positive association between serum folate levels and pulmonary function in children and adults^19^. Therefore, the results of this study suggests that folate may play a role in improving pulmonary function in elderly Korean men.

Several explanations can be considered for the biological mechanisms by which folate improves pulmonary function or prevents pulmonary function from decreasing. Folate is known to have antioxidant activity, removes free radicals, and is associated with oxidative stress-induced apoptosis^20^. This antioxidant activity plays a role in preventing the deterioration of pulmonary function by lowering the pathway inflammation associated with pathogenesis in COPD patients^21^. Furthermore, higher serum folate levels have been reported to play a role in lowering natural killer cell cytotoxicity, which is potentially a biological mechanism^22^. In addition, folate inhibits the expression of major immunomodulatory genes by promoting DNA methylation and plays several roles related to cell functions associated with the pathogenesis of allergic sensitization^23^.

Also, we analyzed determinants such as age, alcohol, smoking history, height, and weight known to be associated with pulmonary function^12,13^. As a result, in FVC, which represents restrictive ventilation disorder, age was negative determinant, whereas height was positive determinant. FEV1, FEV1/FVC, and FEF 25–75% representing obstructive ventilation disorder were negatively correlated with smoking history. Alcohol history did not show any association with pulmonary function in this study.

When we measure the effect size using F-squared (ƒ^2^). In the multivariate linear regression analysis with each lung function test result as a dependent variable, the ƒ^2^ in all analysis were 0.35 or more which means a large effect size. This is thought to have resulted from the inclusion of factors related to lung function such as previously known age, height, weight, and smoking history. However, in the unadjusted linear regression analysis, the F value was around 0.002 to 0.02 between each vitamin and pulmonary function test (data not shown), indicating that the effect size was very small between serum vitamins concentration and lung function even though in case with *p* < 0.05.

There is a positive association between vitamin E and pulmonary function; one study reported that increased vitamin E consumption in COPD patients prevented COPD-related mortality^24^. Another study reported that vitamin E intake was positively associated with pulmonary functions such as FEV1 and FVC in adults^25^. However, the results of this study suggest that serum vitamin E value has a negative association with FEV1 and FVC concerning pulmonary function only in men. There may be several reasons for the contrasting results. First, previous studies measured the intake of vitamin E without directly measuring the level of serum vitamin E, whereas this study directly measured the level of serum vitamin E. Second, unlike previous studies targeting adults, this study included the elderly as subjects. Hence, it is difficult to compare the results directly with existing studies, and additional longitudinal research is required.

As far as we know, this is the first large-scale study to examine the relationship between pulmonary function and serum folate, vitamin A, and vitamin E in the elderly population of Korea as per the data available that represents the status of the country population. In addition, in this study, the association between pulmonary function and folate levels in the elderly were compared after excluding subjects with other underlying diseases. Furthermore, the number of subjects was relatively higher than that of other studies, and the serum samples and pulmonary function parameters of the subjects were measured reliably.

Despite these strengths, this study has several limitations. First, we could not include other variables including other vitamins which might be associated with pulmonary function due to the limitations of retrospective studies. Second, due to the nature of the cross-sectional study design, the serum levels of folate, vitamin A, and vitamin E do not represent the long-term status of the subject. Therefore, cohort research and/or analysis at multiple time point are needed for concrete conclusion. Third, the effect size measure showed that the effect of vitamin on lung function was very small as mentioned before. Therefore, further study is needed on the clinical meaning of this result, even though it was statistically significant. Finally, it is essential to investigate the biological mechanisms through which folate improves the pulmonary function.

## Conclusion

We confirmed a significantly positive correlation between serum folate and FEV1, FEV1/FVC, and FEF 25–75% in elderly Korean men over 60 years of age. Therefore, it can be deduced that serum folate plays an important role in pulmonary function in older men in Korea. Future research is needed on the longitudinal effect of folate and the biological mechanisms of its action on pulmonary function.

## Materials and methods

### Study population

**Figure 2:**
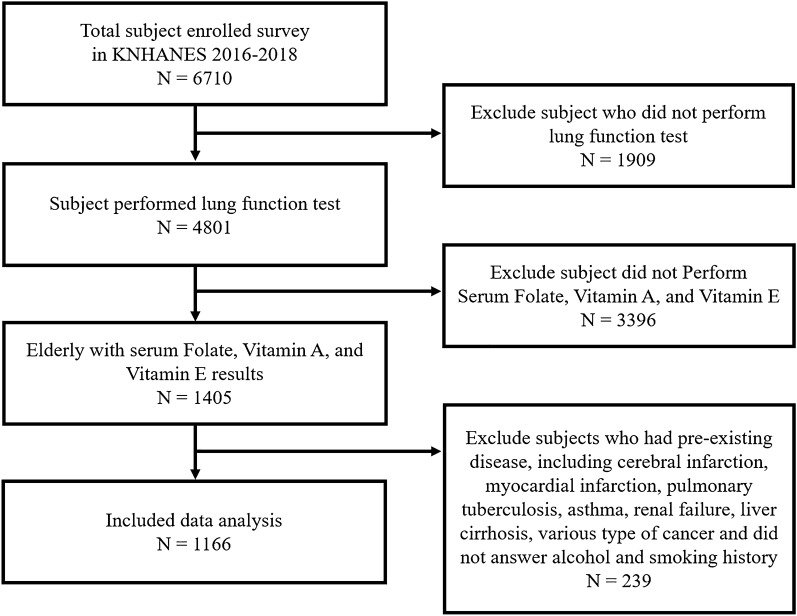
Flowchart of study participants. Among the 6710 participants in the Korea National Health and Nutrition Examination Survey from 2016 to 2018, 4801 participants who underwent pulmonary function tests were considered. Finally, a total of 1166 subjects, excluding those who met the exclusion criteria, were analyzed in this study.

The KNHANES is designed to establish national health policies and conduct nationwide surveys and tests annually to produce national representation and reliability statistics. KNHANES consists of demographic characteristics, chronic disease prevalence, food and nutritional intake, health surveys, and various medical examinations. Detailed information about this study was provided to subjects or legal guardians and informed consent was obtained from all enrolled subjects. We analyzed the association between folate, vitamin A, and vitamin E levels and pulmonary function in the elderly aged 60–80 years from the KNHANES data from 2016 to 2018. Among 6710 subjects, 1,909 patients who were not tested for pulmonary function were first excluded, and 3396 patients who were not tested for vitamin A, vitamin E, and folate were subsequently excluded. The following subjects with one or more underlying diseases were excluded from the study: 50 patients with stroke, 85 with myocardial infarction and angina, 81 with pulmonary tuberculosis, 56 with asthma, 6 with renal failure, 14 with liver cirrhosis, and patients with various cancers (15 stomach cancer, 5 liver cancer, 17 colorectal cancer, 11 breast cancer, 10 cervical cancer, 5 lung cancer, 12 thyroid cancer, and 28 other cancer patients). Further, those who did not disclose their drinking or smoking history were excluded. Finally, 1166 subjects (528 men and 637 women) were enrolled for the analysis. (Fig. 2). All methods and protection of personal information were performed in accordance with the Declaration of Helsinki.

### Measurements of variables

Subjects were assessed on their alcohol consumption and smoking history through a self-survey, and height and weight measurements were performed. Vitamin A and vitamin E serum levels were measured using the high-performance liquid chromatography-fluorescence detection (HPLC-FID) test method, with Agilent 1200 (Agilent, Santa Clara, CA, USA) test equipment, and Chromsystems (Chromsystems Instruments & Chemicals, Gräfelfing, Germany) reagents. The adult reference ranges for serum vitamin A and vitamin E were considered to be 0.30–0.70 (mg/L) and 5.00–20.00 (mg/L), respectively. Serum folate levels were measured using ARCHITECT i4000Sr (Abbott, Abbott Park, IL, USA) test equipment and ARCHITECT Folate reagent (Abbott, Abbott Park, IL, USA) using the Chemiluminescent Microparticle Immunoassay (CMIA method). The reference range of serum folate was 3.1–20.5 (ng/mL). Pulmonary function tests (PFTs) were performed using a pulmonary function test device with disposable filters (Vyntus™ Spiro; CareFusion, San Diego, CA, USA) by experienced examiners. All pulmonary function tests were performed without bronchodilator use. We measured the following parameters by pulmonary function tests: forced vital capacity (FVC); refers to the total expiratory volume exhaled with one breath during the maximum effort expiration, forced expiratory volume in one second (FEV1); refers to the expiratory volume expelled in the first second of the maximum effort expiration and is associated with the severity of obstructive ventilation disorder, forced expiratory volume in one second/forced vital capacity (FEV1/FVC), forced expiratory flow at 25% and 75% of the pulmonary volume (FEF25–75%); the mean forced expiratory flow during the middle half of the FVC, which helps diagnose peripheral small airway obstruction and is the first abnormal finding in smokers, forced expiratory volume in 6 s (FEV6), and peak expiratory velocity (PEV).

### Statistical analysis

We used the chi-square test for comparative analysis based on the alcohol consumption status, while the Student’s t-test analysis was performed to compare each variable between men and women. A linear-by-linear association test was performed for comparative analysis based on the amount of smoking. Regression analysis was performed by considering FEV1, FVC, FEF25–75%, FEV1/FVC, PEV, and FEV6 as dependent variables to evaluate the effect of each item on each pulmonary function value. In addition, Cohen's D in t-test result and F-squared values in linear regression analysis were used to measure the effect size. And, the method used is described in the supplementary file. (Supplemental method) We used SPSS statistical software package version 17 (SPSS Inc., Chicago, USA) for all analyses and a *p* value < 0.05 was considered statistically significant.

### Ethics statement

This research study was conducted retrospectively from data obtained for clinical purposes. We consulted extensively with the Institutional Review Board of Jeju National University Hospital who determined that our study did not need ethical approval. An IRB official waiver of ethical approval was granted from the Institutional Review Board of Jeju National University Hospital.

## Supplementary Information

Supplementary Information 1.

Supplementary Information 2.

## Data Availability

All available data generated or analyzed during this study are included in this published article. Other raw data are not available because of regulation of data sharing in the Republic of Korea.
