# Supplementary material for: High serum folate level is positively associated with pulmonary function in elderly Korean men, but not in women
Source: Sci Rep. 2022 Mar 16;12:4523. doi: 10.1038/s41598-022-08234-9 (PMC8927119; doi:10.1038/s41598-022-08234-9)
Supplement: Supplementary file 1 — Supplementary Information 1. [file 41598_2022_8234_MOESM1_ESM.docx]

Cohen’s D test is used to analyze the effect size in the comparison between men and women. And, |d| = 0.20, 0.50, and 0.80 indicate the cut-off value for a small, medium, and large effect, respectively.

Cohen’s D (|d|) is calculated as $D=\frac{M_{1}-M_{2}}{S_{p}}$

The pooled standard deviation ($S_{p}$) is calculated as $S_{p}=\sqrt{\frac{\left( n_{1}-1 \right){S_{1}}^{2}+\left( n_{2}-1 \right){S_{2}}^{2}}{n_{1}+n_{2}-2}}$

$M_{1}$ and $M_{2}$: the means for groups

$n_{1}$ and $n_{2}$: number of groups

$S_{1}$and $S_{2}$: standard deviation for groups

F-squared (ƒ^2^) is used to measure the effect size of linear regression model. ƒ^2^ is calculated below and $R_{inc}^{2}$ denotes the increase in r-square for a set of predictors over another set of predictors.

$$ƒ2=\frac{R_{inc}^{2}}{1-R_{inc}^{2}}$$

ƒ^2^ indicate a cut-off value at 0.02, 015, and 0.35 as a small, medium, and large effect, respectively.
