# Supplementary material for: High serum folate level is positively associated with pulmonary function in elderly Korean men, but not in women
Source: Sci Rep. 2022 Mar 16;12:4523. doi: 10.1038/s41598-022-08234-9 (PMC8927119; doi:10.1038/s41598-022-08234-9)
Supplement: Supplementary file 2 — Supplementary Information 2. [file 41598_2022_8234_MOESM2_ESM.docx]

Supplemental table 1. Unadjusted linear regression analysis between serum vitamin concentration and pulmonary function test in all subjects

|  | Folate | | Vitamin A | | Vitamin E | |
| --- | --- | --- | --- | --- | --- | --- |
|  | *r* | p | *r* | p | *r* | p |
| FVC | ***-0.150*** | ***<0.001*** | ***0.119*** | ***<0.001*** | ***-0.099*** | ***0.001*** |
| FEV1 | ***-0.080*** | ***0.006*** | ***0.108*** | ***<0.001*** | ***-0.069*** | ***0.019*** |
| FEV1/FVC | ***0.161*** | ***<0.001*** | -0.033 | 0.263 | ***0.068*** | ***0.020*** |
| FEV6 | ***-0.130*** | ***<0.001*** | ***0.117*** | ***<0.001*** | ***-0.093*** | ***0.001*** |
| FEF25-75% | 0.052 | 0.077 | 0.041 | 0.158 | 0.023 | 0.425 |
| PEV | -0.056 | 0.055 | ***0.127*** | ***<0.001*** | ***-0.068*** | ***0.020*** |

*r*: Pearson correlation coefficient;

Bold italics indicates statistical significance (p<0.05)

Supplemental table 2. Unadjusted linear regression analysis between serum vitamin concentration and pulmonary function test in men

|  | Folate | | Vitamin A | | Vitamin E | |
| --- | --- | --- | --- | --- | --- | --- |
|  | *r* | p | *r* | p | *r* | p |
| FVC | 0.044 | 0.312 | -0.037 | 0.401 | ***-0.096*** | ***0.028*** |
| FEV1 | ***0.100*** | ***0.021*** | 0.008 | 0.860 | -0.061 | 0.164 |
| FEV1/FVC | ***0.119*** | ***0.006*** | 0.074 | 0.090 | 0.031 | 0.476 |
| FEV6 | 0.069 | 0.112 | -0.023 | 0.597 | ***-0.086*** | ***0.047*** |
| FEF25-75% | ***0.123*** | ***0.005*** | 0.049 | 0.261 | 0.008 | 0.858 |
| PEV | ***0.128*** | ***0.003*** | 0.070 | 0.109 | -0.019 | 0.657 |

*r*: Pearson correlation coefficient;

Bold italics indicates statistical significance (p<0.05)

Supplemental table 3. Unadjusted linear regression analysis between serum vitamin concentration and pulmonary function test in women

|  | Folate | | Vitamin A | | Vitamin E | |
| --- | --- | --- | --- | --- | --- | --- |
|  | *r* | p | *r* | p | *r* | p |
| FVC | 0.035 | 0.374 | -0.017 | 0.664 | 0.064 | 0.104 |
| FEV1 | 0.039 | 0.332 | -0.009 | 0.815 | 0.064 | 0.107 |
| FEV1/FVC | 0.015 | 0.712 | 0.016 | 0.686 | 0.013 | 0.739 |
| FEV6 | 0.038 | 0.341 | -0.015 | 0.704 | 0.061 | 0.127 |
| FEF25-75% | 0.004 | 0.918 | 0.017 | 0.668 | 0.049 | 0.212 |
| PEV | ***0.095*** | ***0.017*** | -0.064 | 0.107 | 0.026 | 0.511 |

*r*: Pearson correlation coefficient;

Bold italics indicates statistical significance (p<0.05)

Supplemental Table 4. Multivariate linear regression analysis considering PEV as dependent variable

|  | Total | | | Men | | | Women | | |
| --- | --- | --- | --- | --- | --- | --- | --- | --- | --- |
|  | B | 95% CI | p | B | 95% CI | p | B | 95% CI | p |
| Sex | ***-1.451*** | ***-1.776 ~ -1.116*** | ***<0.001*** |  |  |  |  |  |  |
| Age | ***-0.081*** | ***-0.095 ~ -0.067*** | ***<0.001*** | ***-0.098*** | ***-0.125 ~ -0.072*** | ***<0.001*** | ***-0.065*** | ***-0.079 ~ -0.051*** | ***<0.001*** |
| Alcohol Hx | -0.075 | -0.290 ~ 0.141 | 0.496 | 0.107 | -0.477 ~ 0.690 | 0.720 | -0.074 | -0.251 ~ 0.102 | 0.408 |
| Smoking Hx | 0.218 | -0.032 ~ 0.467 | 0.088 | 0.181 | -0.187 ~ 0.549 | 0.333 | 0.289 | -0.055 ~ 0.633 | 0.100 |
| Height | ***0.046*** | ***0.029 ~ 0.062*** | ***<0.001*** | ***0.040*** | ***0.008 ~ 0.071*** | ***0.013*** | ***0.053*** | ***0.037 ~ 0.069*** | ***<0.001*** |
| Weight | ***0.033*** | ***0.022 ~ 0.044*** | ***<0.001*** | ***0.045*** | ***0.025 ~ 0.066*** | ***<0.001*** | ***0.017*** | ***0.006 ~ 0.028*** | ***0.002*** |
| Folate | ***0.038*** | ***0.015 ~ 0.060*** | ***0.001*** | ***0.072*** | ***0.027 ~ 0.118*** | ***0.002*** | 0.016 | -0.005 ~ 0.037 | 0.126 |
| Vitamin A | 0.190 | -0.257 ~ 0.638 | 0.404 | 0.205 | -0.281 ~ 1.239 | 0.216 | -0.292 | -0.770 ~ 0.186 | 0.231 |
| Vitamin E | -0.010 | -0.025 ~ 0.006 | 0.210 | -0.027 | -0.058 ~ 0.005 | 0.094 | 0.001 | -0.013 ~ 0.015 | 0.891 |

CI: confidence interval

Bold italics indicates statistical significance (p<0.05)

Supplemental Table 5. Multivariate linear regression analysis considering FEV6 as dependent variable

|  | Total | | | Men | | | Women | | |
| --- | --- | --- | --- | --- | --- | --- | --- | --- | --- |
|  | B | 95% CI | p | B | 95% CI | p | B | 95% CI | p |
| Sex | ***-0.417*** | ***-0.518 ~ -0.316*** | ***<0.001*** |  |  |  |  |  |  |
| Age | ***-0.031*** | ***-0.035 ~ -0.026*** | ***<0.001*** | ***-0.039*** | ***-0.047 ~ -0.032*** | ***<0.001*** | ***-0.026*** | ***-0.030 ~ -0.021*** | ***<0.001*** |
| Alcohol Hx | 0.050 | -0.017 ~ 0.116 | 0.145 | 0.156 | -0.014 ~ 0.325 | 0.073 | 0.048 | -0.011 ~ 0.107 | 0.112 |
| Smoking Hx | -0.016 | -0.057 ~ -0.025 | 0.445 | -0.016 | -0.073 ~ 0.041 | 0.577 | -0.008 | -0.070 ~ 0.053 | 0.787 |
| Height | ***0.043*** | ***0.038 ~ 0.048*** | ***<0.001*** | ***0.055*** | ***0.045 ~ 0.064*** | ***<0.001*** | ***0.034*** | ***0.029 ~ 0.040*** | ***<0.001*** |
| Weight | 0.000 | -0.004 ~ 0.003 | 0.871 | -0.001 | -0.007 ~ 0.005 | 0.761 | -0.003 | -0.007 ~ 0.001 | 0.120 |
| Folate | 0.002 | -0.005 ~ 0.009 | 0.503 | 0.013 | 0.000 ~ 0.026 | 0.055 | -0.003 | -0.010 ~ 0.004 | 0.405 |
| Vitamin A | -0.032 | -0.170 ~ 0.107 | 0.652 | -0.099 | -0.320 ~ 0.123 | 0.382 | -0.012 | -0.172 ~ 0.149 | 0.888 |
| Vitamin E | -0.003 | -0.008 ~ 0.001 | 0.168 | ***-0.012*** | ***-0.021 ~ -0.003*** | ***0.009*** | 0.002 | -0.003 ~ 0.007 | 0.358 |

CI: confidence interval

Bold italics indicates statistical significance (p<0.05)
